# Use of Calcium Modification in Percutaneous Coronary Intervention: A Comprehensive Review

**DOI:** 10.3390/jcm14228130

**Published:** 2025-11-17

**Authors:** Noyan Ramazani, Ala W. Abdallah, Michael V. DiCaro, Divyansh Sharma, Aditi Singh, KaChon Lei

**Affiliations:** 1 Department of Internal Medicine, Kirk Kerkorian School of Medicine at University of Nevada, Las Vegas, NV 89119, USA; michael.dicaro@unlv.edu (M.V.D.); aditi.singh@unlv.edu (A.S.); 2 Department of Internal Medicine, Carver College of Medicine, University of Iowa, Iowa City, IA 52242, USA; ala-abdallah@uiowa.edu; 3 Department of Cardiovascular Medicine, Kirk Kerkorian School of Medicine at University of Nevada, Las Vegas, NV 89119, USA; divyansh.sharma@unlv.edu

**Keywords:** calcified coronary lesions, percutaneous coronary intervention, atherosclerotic plaque remodeling, coronary CT angiography, positron emission tomography, photon-counting CT, dual-energy CT, balloon escalation technique, intravascular lithotripsy, rotational atherectomy, orbital atherectomy, laser atherectomy

## Abstract

Calcified coronary lesions remain a challenge in percutaneous coronary intervention (PCI) in both situations of acute myocardial infarction (MI) and stable coronary syndrome. It significantly increases the risk of procedural complications due to difficulty in equipment delivery, balloon expansion, and stent delivery. Furthermore, stent thrombosis, dissection, perforation, and future in-stent restenosis occur more frequently in calcified coronary lesions, impacting repeat target vessel revascularization and increasing the risk of future MI. With intracoronary imaging (intravascular ultrasound and optical coherence tomography), peri-procedural success for treating calcified lesions has increased significantly. Different modalities of calcium modification techniques have since been introduced. This review will discuss the pathophysiology and phenotypes of calcium deposition in the coronary vessels, including eccentric calcified plaques and calcified nodules. We will also focus on calcium modification techniques and their mechanisms: (1) Balloon escalation technique, (2) intravascular lithotripsy, (3) orbital atherectomy, and (4) rotational atherectomy. We will focus on the strengths and limitations of each technique, based on current recommendations and expert consensus from SCAI. We will also provide contemporary evidence of each modality for treating different phenotypes of calcified lesions. In summary, this article provides a comprehensive review of current guidelines for optimizing the treatment of calcified coronary lesions in PCI.

## 1. Introduction/Background

Acute coronary syndrome (ACS) involves a sudden reduction in blood perfusion and, consequentially, oxygen supply to the cardiomyocytes and includes ST-segment elevation myocardial infarction (STEMI), non-STEMI (NSTEMI), and unstable angina [1]. STEMI patients often have and present with multi-vessel coronary artery disease (mvCAD), and percutaneous coronary intervention (PCI) represents the undisputed treatment modality in such cases [2]. ACS is caused by the physical blockage or narrowing of coronary arteries, either transiently by arterial spasms due to extreme temperature ranges, stress, or medications and illicit drug use, or as chronic issues such as atherosclerosis complicated in the setting of diabetes, hypertension, dyslipidemia, tobacco use disorder, metabolic syndrome, and obesity. Genetic predispositions, such as a family history of heart disease, can also contribute to poor patient outcomes and place adverse risks on otherwise healthy patients.

Based on the 2025 American College of Cardiology (ACC), American Heart Association (AHA), and the Society for Cardiovascular Angiography and Intervention (SCAI) guidelines for the management of patients with ACS, there is an indication for emergent revascularization via PCI in patients with significant myocardium dysfunction, such as those in fulminant cardiogenic shock, with secondary prevention guidelines including lipid management and cardiac rehabilitation after hospital discharge [3]. Based on the guidelines of the AHA and ACC, the typical goal for door-to-balloon (D2B) time is 90 min or less for patients to receive a PCI due to complications arising from a STEMI, in order to help restore blood flow to the cardiomyocytes and decrease mortality [3].

Calcified coronary artery lesions represent a significant barrier to the success of PCI, particularly in patients with acute MI or chronic stable angina. These lesions pose multiple obstacles to the success of PCI, including limitations in equipment delivery, reduced balloon and stent expansion, and increased likelihood of intraprocedural complications such as dissection and perforation, as well as postprocedural complications like stent thrombosis or restenosis [4]. As a result, calcified coronary lesions are associated with higher rates of target vessel revascularization and long-term major adverse cardiovascular events (MACE), including recurrent MI.

Coronary artery calcification (CAC), the process of calcium deposition that forms these lesions, is a hallmark of advanced atherosclerotic disease and is frequently observed in older patients, those with diabetes mellitus (DM), and individuals with chronic kidney disease (CKD). Studies in the literature show that coronary artery calcification is present in up to 50% of patients undergoing PCI, with severe calcification observed in 10–25% of cases [5]. A meta-analysis, including a total of 113,306 patients, that was conducted to study the prevalence of CAC in different demographics, concluded that CAC was more prevalent and statistically significant in male patients, those of older age, patients with diabetes, and those with hypertension [6].

The prevalence is higher in patients with multiple cardiovascular risk factors. Cardiovascular risk factors that aid in the development and progression of coronary artery calcification include traditional cardiovascular risk factors such as age, hypertension, hyperlipidemia, DM, and smoking. Systemic conditions, such as CKD, amplify the risk of coronary artery calcification due to altered mineral metabolism, including complications related to hyperphosphatemia and secondary hyperparathyroidism due to the poor excretion function of the kidneys [7].

Patients with calcified coronary lesions often present with symptoms of chest pain, dyspnea, or anginal equivalents. These presentations span a spectrum from stable angina to acute coronary syndromes (ACS), including non-ST-elevation MI (NSTEMI) and ST-elevation MI (STEMI). Fixed calcified coronary lesions often cause a stable angina presentation by restricting blood flow during exertion. ACS is typically associated with plaque disruption, such as rupture of the calcified nodules, which can trigger thrombus formation. Calcified lesions are linked to worse clinical outcomes in both stable and unstable coronary syndromes [7].

## 2. Materials and Methods

We conducted a narrative review using PubMed, Embase, and Google Scholar research databases to identify contemporary consensus statements, pertinent trials, and registries on calcium modification in percutaneous coronary intervention (PCI) along with supplemental material surrounding coronary artery disease (CAD), calcium deposits, calcium coronary lesions, eccentric calcified plaques, calcified nodules, and calcium modification techniques. An emphasis on diagnostic imaging modalities and mechanistic therapeutic approaches with device utilization to decrease calcium burden was investigated, with most of the literature dated within the last 10 years.

## 3. Pathophysiology of Calcium Deposition in Coronary Vessels

Coronary artery calcification is an active, regulated process that has been suggested in the literature to resemble bone formation, rather than merely a passive calcium buildup. Vascular smooth muscle cells (VSMCs) play a major role in this process, which begins with endothelial injury or chronic stress, triggering inflammation and oxidative stress. These signals induce the expression of bone-associated proteins in VSMCs, transforming them into osteoblast-like cells capable of producing a calcified extracellular matrix [8]. The process of transforming VSMCs into osteoblast-like cells is referred to as osteogenic differentiation. It is regulated by molecular pathways like bone formation, influenced by inflammation, oxidative stress, lipid accumulation, and apoptosis.

**Figure 1:**
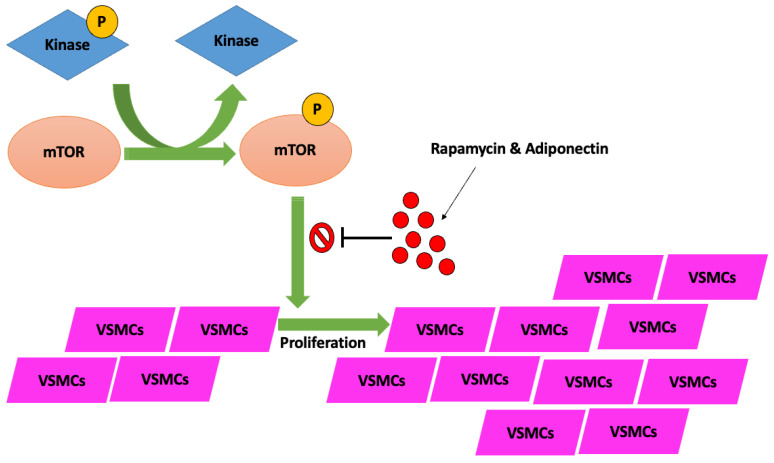
Cellular pathway of mTOR phosphorylation by kinase-specific enzymes, subsequent deactivation of mTOR-Phos complex by rapamycin and adiponectin mediators, which prevent VSMC proliferation. Lack of VSMC activation and subsequent proliferation by the mTOR-Phos complex prevents downstream complications related to the creation of atherosclerotic plaque and vascular calcification, and stenosis. P = phosphate; mTOR = mechanistic target of rapamycin; VSMCs = vascular smooth muscle cells.

These mechanisms converge to promote calcium deposition in the arterial wall, resulting in increased arterial stiffness and clinical complications, including atherosclerosis and vascular dysfunction. Research on the mammalian target of rapamycin (mTOR) signaling pathways has also shown the vital role of mTOR in osteoblastic differentiation through the transformation of VSMCs, where therapies such as rapamycin and protein hormones like adiponectin in rats have played a vital role in inhibiting vascular calcification through the regulation of the mTOR cell signaling pathway (Figure 1) [8,9].

**Figure 2:**
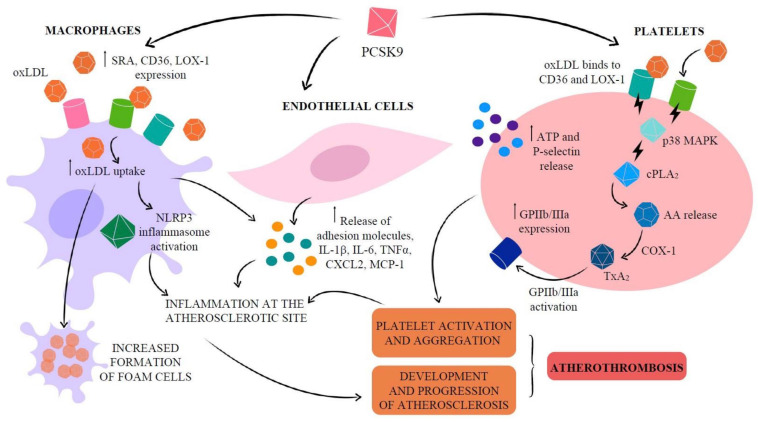
Biochemical and molecular pathways implicated in atherosclerotic and calcified plaque genesis with specific emphasis on the impact of PCSK9 and oxLDL. LDL: low-density lipoprotein; oxLDL: oxidized LDL; LOX-1: lectin-like oxidized low-density lipoprotein receptor 1; SRA: scavenger receptor A; CD36: cluster differentiation 36; NLRP3: NOD-like receptor pyrine domain-containing proteins 3; PLA_2_: phospholipase A_2_; AA: arachidonic acid; COX: cyclooxygenase; TX: thromboxane; GPIIb/IIIa: glycoprotein IIb/IIIa. Source: Barale, C.; Melchionda, E.; Morotti, A.; Russo, I. PCSK9 biology and its role in atherothrombosis. *Int. J. Mol. Sci.* **2021**, *22*, 5880.

Osteogenic differentiation is a process in which VSMCs lose their contractile phenotype and acquire osteoblast-like properties. Key contributors to vascular calcification caused by osteogenic differentiation include pro-inflammatory cytokines, oxidized low-density lipoprotein (oxLDL), reactive oxygen species (ROS), autotaxin (ATX), and proprotein convertase subtilisin/kexin type 9 (PCSK9), which is involved in lipoprotein metabolism and has been shown to have a stimulatory impact and crucial role in vascular calcification, thereby complicating atherosclerosis (Figure 2) [10].

The calcification process is also facilitated by the release of matrix vesicles and apoptotic bodies, which act as nucleation sites for calcium phosphate crystal deposition. Pro-inflammatory cytokines, such as tumor necrosis factor-alpha (TNF-α), interleukin-1β (IL-1β), and interleukin-6 (IL-6) in general, stimulate the expression of bone-related proteins such as alkaline phosphatase (ALP), runt-related transcription factor 2 (RUNX2), osteocalcin, and bone morphogenetic proteins (BMPs), which are important regulators of the vascular calcification process [10,11].

TNF-α plays a significant role by activating the nuclear factor-kappa B (NF-κB) pathway, a key transcription factor in inflammation. NF-κB upregulates the expression of RUNX2, ALP, and osteocalcin [12]. TNF-α also enhances the release of matrix vesicles from VSMCs, which are an independent facilitator of the vascular calcification process. Elevated TNF-α levels have been linked to the severity of vascular calcification in atherosclerosis and inflammatory diseases such as rheumatoid arthritis.

IL-1β amplifies calcification through oxidative stress and mitochondrial dysfunction. It upregulates the expression of BMP-2 and BMP-4 and increases ALP activity [11,13]. IL-1β synergizes with TNF-α to further escalate inflammatory signaling. IL-1β also induces apoptosis of VSMCs, releasing apoptotic bodies that serve as an independent stimulator of osteogenic differentiation and vascular calcification.

IL-6 is a potent cytokine that activates the Janus kinase/signal transducer and activator of transcription (JAK/STAT) pathway. This activation promotes the expression of RUNX2 and BMPs, particularly BMP-2 [13]. Elevated levels of IL-6 have been strongly associated with increased vascular stiffness and calcification in conditions such as chronic kidney disease and diabetes.

Oxidative stress is a major contributing factor to VSMCs’ osteogenic differentiation. Through ROS, generated during oxidative stress, they activate pathways such as the NF-κB pathway by degrading the inhibitor of NF-κB (IκB). Activation of NF-κB leads to the production of ALP and RUNX2. ROS also activates the mitogen-activated protein kinase (MAPK) pathway, particularly p38 MAPK signaling, which also stimulates the production of ALP and RUNX3 [14]. ALP has been shown to facilitate vascular calcification through the micro-restructuring of coronary plaques while inciting systemic inflammation, like the chronic exposure of hazardous airborne pollutants, including fine particulate matter (PM_2.5_), polycyclic aromatic hydrocarbons (PAHs), and heavy metals, all of which increase the risk of CAC [15,16].

The Wnt/β-catenin pathway is similarly influenced by ROS; they stabilize β-catenin, a key protein in this signaling cascade. Activated β-catenin drives the production of bone matrix proteins like osteocalcin and osteopontin, which are essential for calcification, while also strengthening RUNX2 activity. Also, ROS stimulates the Phosphoinositide 3-kinase/Akt pathway, which supports cellular growth and differentiation. This pathway boosts matrix vesicle production, increases ALP activity, and enhances RUNX2 expression, reinforcing the osteogenic transformation of VSMCs [14]. ROS also contributes to vascular calcification through apoptosis of VSMCs, a process that releases apoptotic bodies into the vessel wall.

Lipid accumulation, particularly oxLDL, plays a central role in vascular calcification by driving the transformation of VSMCs into bone-like cells [17]. OxLDL is formed through the oxidative modification of LDL in areas of endothelial dysfunction and initiates a cascade of inflammatory and oxidative processes [17]. OxLDL binds to receptors such as lectin-type oxidized LDL receptor 1 (LOX-1) and Toll-like receptors (TLRs) on VSMCs, activating the release of pro-inflammatory cytokines like IL-1β and TNF-α [10]. These cytokines stimulate the osteogenic transformation of VSMCs by increasing the expression of RUNX2, a critical factor in bone formation processes. OxLDL generates ROS, amplifying oxidative stress, which promotes VSMC osteogenic differentiation, as discussed earlier. OxLDL facilitates the release of matrix vesicles from VSMCs.

Matrix vesicles are extracellular, membrane-bound particles released by VSMCs that play a crucial role in the early stages of vascular calcification. These vesicles are enriched with calcium and phosphate ions, as well as osteogenic enzymes such as ALP, annexins, and nucleotidases [18]. Matrix vesicles serve as nucleation sites for hydroxyapatite crystal formation, a process essential for the progression of calcification. The release of matrix vesicles is triggered by various stimuli, including mechanical stress, oxidative stress, and inflammatory cytokines [18]. Within these vesicles, enzymes such as ALP degrade inhibitors of calcification, such as pyrophosphate, while annexins and phosphatidylserine provide a scaffold for calcium–phosphate complex formation. This process is tightly regulated, and its dysregulation leads to pathological calcification.

Apoptosis of VSMCs is a critical driver of vascular calcification. During apoptosis, VSMCs lose their structural integrity and release apoptotic bodies into the extracellular space [19]. These apoptotic bodies, which contain cellular debris and vesicles rich in calcium and phosphate, serve as additional nucleation sites for hydroxyapatite crystal deposition. Apoptosis-induced calcification is amplified by the loss of natural inhibitors such as matrix Gla protein, which usually binds to calcium and prevents its deposition [19]. This process is particularly prominent in advanced atherosclerotic plaques, where chronic inflammation and oxidative stress accelerate VSMC apoptosis. As apoptotic bodies accumulate, they contribute to the stiffness and instability of calcified lesions, increasing the risk of plaque rupture and cardiovascular events.

Bone-associated proteins that were mentioned earlier are crucial regulators of vascular calcification, mirroring the processes observed in bone formation. Among these proteins, ALP plays a central role by degrading pyrophosphate, a natural inhibitor of calcification. Pyrophosphate binds to calcium and prevents its crystallization; ALP hydrolyzes this molecule, thus creating a local environment conducive to hydroxyapatite crystal formation. Elevated ALP activity in VSMCs is a hallmark of osteogenic differentiation, directly contributing to the mineralization of the vascular wall.

Other bone-associated proteins, such as osteopontin and osteocalcin, further mediate the calcification process. Osteopontin is a protein that regulates the size and organization of calcium deposits by binding to hydroxyapatite crystals and stabilizing their structure. Osteocalcin, produced by VSMCs during osteogenic differentiation, interacts with calcium ions in the extracellular matrix, enhancing the rigidity and stability of calcified lesions.

## 4. Atherosclerotic Development Timeline

### 4.1. Adaptive and Pathologic Intimal Thickening

**Figure 3:**
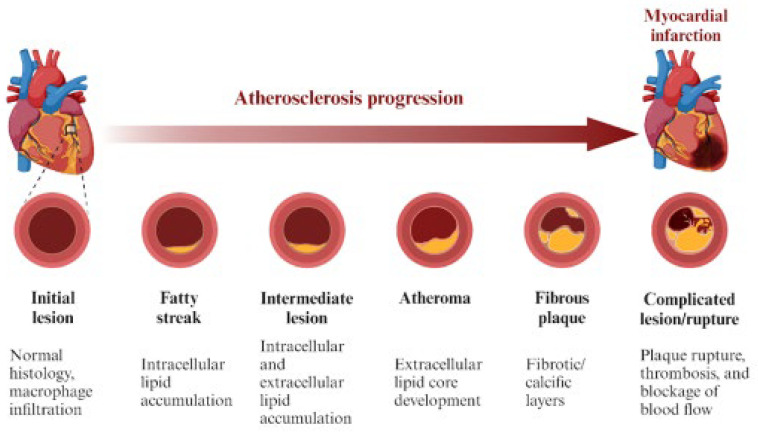
The progression of atherosclerosis from initial lesion morphology to final complicated plaque formation, rupture, and thrombosis, causing MI. MI = myocardial infarction. Source: Florek, K.; Bartoszewska, E.; Biegała, S.; Klimek, O.; Malcharczyk, B.; Kübler, P. Rotational atherectomy, orbital atherectomy, and intravascular lithotripsy comparison for calcified coronary lesions. *J. Clin. Med.* **2023**, *12*, 7246.

Intimal thickening can be classified into physiological or adaptive intimal thickening (AIT), also known as “intimal masses,” and pathologic intimal thickening (PIT). AIT consists of several layers of smooth muscle cells (SMCs). It is in reference to the byproduct of neonatal intimal formation commonly seen in the left anterior descending (LAD) coronary artery, which is rarely formed before 30 weeks of gestation but seen 3 months after birth with apparent thickening in the tunica intima layer of the LAD [20]. The SMCs in AIT can differentiate into macrophages, which can later be prone to lipid accumulation and, ultimately, form intimal xanthomas or fatty streak lesions (Figure 3) [21]. Coronary artery atherosclerosis transforming to calcification with further progression to plaque formation in the setting of luminal narrowing is often absent or minimal in early lesions, such as in AIT and PIT.

PIT is a combination of lipid pools (LP), which are the creation of extracellular lipid deposits and proteoglycans (e.g., versican and hyaluronan), and layers of SMCs, which ultimately leads to the creation of microcalcifications or punctate calcifications within the intimal layer and the construction of fatty streaks pointing to plaque progression [22,23].

Past research has investigated the high affinity of LP for retaining plasma lipoproteins in addition to the amplification of proteoglycans such as biglycan and decorin in the early construction of fatty streaks, which showed high signaling preference for apo B and fibrinogen, the former being a common component of low-density lipoproteins (LDLs) [24,25,26]. 

These microcalcifications are believed to be the result of apoptosis of SMCs occurring in the intimal layer and forming at the border of this necrotic core, which initially appears as a speckled pattern and later transforms into fragments [22]. Macrophages are deposited into the LP as matrix metalloproteinases, infiltrating the surrounding area and leading to the development of a necrotic core, which in turn facilitates the formation of the fibroatheroma.

### 4.2. Formation of Fibroatheroma

**Figure 4:**
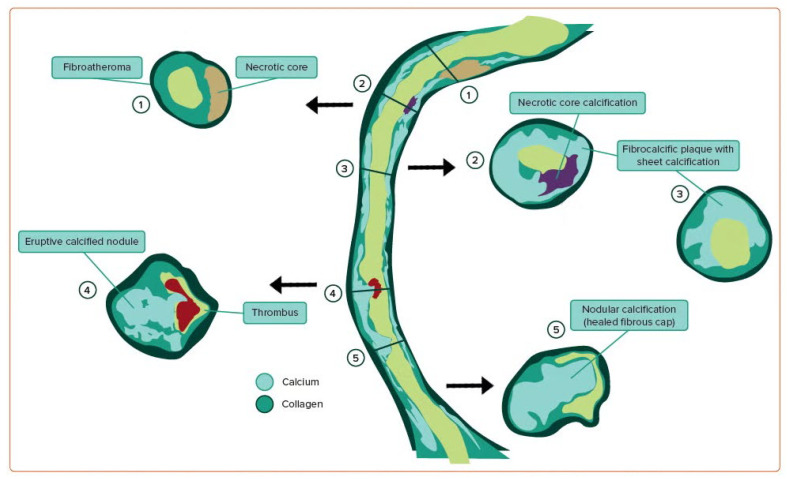
Illustration of the morphological types of coronary artery calcification. Source: Morris, M.C.; Kreutz, R.P. Coronary Calcification: Types, Morphology and Distribution. *Interv. Cardiol.* **2025**, *20*, e13 [22].

If atherosclerosis progresses, then the characteristic fibroatheroma (Figure 4) is formed, which histologically consists of a necrotic core or a non-cellular structural unit caused by the impaired efferocytosis of infiltrated macrophages into the LP and the release of matrix metalloproteinases, causing elevated levels of apoptosis [27]. Early fibroatheroma may contain punctate calcification bodies, whereas late fibroatheroma often contains larger volumes of acellular necrotic cores consistent with macrophage apoptosis and the accumulation of free cholesterol with a small percentage of extracellular matrix [22,23].

A significant upregulation in the G-protein-coupled receptors pathway via Gsα (G-protein stimulatory α subunit) protein occurs during the development of a necrotic core and the creation of a fibroatheroma [22,28]. This is facilitated by the Ox-LDL units causing *Gnas* transcription activation through the activation of ERK1/2 and C/EBPβ phosphorylation via oxidative stress, ultimately leading to foam cell formation and deposition, and, consequently, leading to late-stage atherosclerosis progression [28].

The necrotic core is later combined with an overlying thick fibrous cap to form the fibroatheroma structure. Thick-cap fibroatheroma eventually thins secondarily to the infiltration of macrophages, SMC death (apoptosis), and the lack of new collagen deposition, transforming the thick-cap fibroatheroma into a volatile thin-cap fibroatheroma (TCFA) [27]. Thin-cap fibroatheroma, often referred to as a vulnerable plaque, due to its cap depth of ≤65 µm and its high susceptibility to shearing forces created by blood flow, is often the sole culprit responsible for the cause of myocardial infarction and is implicated in plaque rupture and instability [27].

### 4.3. Plaque Rupture and Plaque Erosion

TCFAs may contain microcalcification bodies or punctate calcific subunits with the possibility of intraplaque hemorrhage (IPH) occurring with exposure between the thrombogenic core and the circulating blood, making the plaque vulnerable to rupture [22]. Recent research has investigated the impact and presence of hemosiderin as a byproduct of the macrophage-induced phagocytosis of hemoglobin near the region of intraplaque hemorrhage, limiting some advanced imaging techniques utilized to examine calcium modification during PCI procedures [29].

Plaque erosion, opposite to plaque rupture, is due to the sustainability of the thick fibrous cap remaining almost intact, while loss of the basement membrane integrity and endothelial cell desquamation occurs [22,30]. Plaque rupture is caused by shear stress gradients and activation of Toll-like receptor 2 (TLR-2) in endothelial cells, with subsequent activation and aggregation of neutrophil extracellular traps (NETs) and thrombosis causing a decrease in morbidity outcomes, when compared to plaque rupture as the predominant mechanism, resulting in a non-ST-segment elevation myocardial infarction (NSTEMI) responsible for approximately 40% of patients with acute coronary syndrome (ACS) [30,31].

### 4.4. Variations in Calcified Plaque Morphology

**Figure 5:**
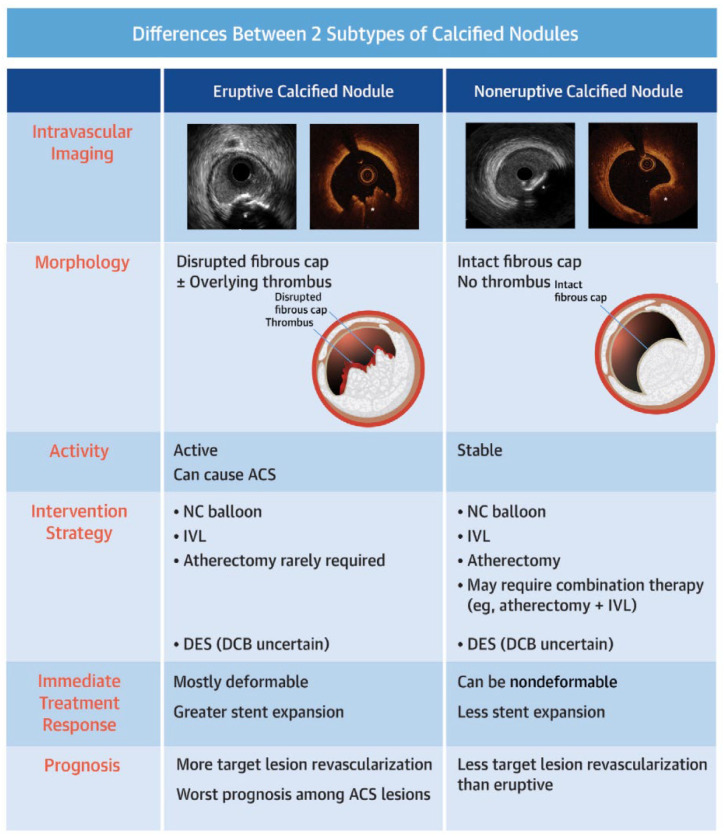
Variations between the 2 major subtypes of calcified nodules frequently seen in percutaneous coronary intervention procedures. ACS = acute coronary syndrome; DCB = drug-coated balloon; DES = drug-eluting stent(s); IVL = intravascular lithotripsy; NC = non-compliant. * = intravascular calcified nodule. Source: Shin, D.; Karimi Galougahi, K.; Spratt, J. C.; Maehara, A.; Collet, C.; Barbato, E., Ribichini, F.L.; Gonzalo, N.; Sakai, K.; Mintz, G.S.; et al. Calcified nodule in percutaneous coronary intervention: therapeutic challenges. *Cardiovasc. Interv.* **2024**, *17*, 1187–1199.

Most acute coronary syndromes (ACS) are caused by plaque rupture or plaque erosion. Still, a subset is due to complicated calcified nodules (CNs), which have emerged as a distinct and highly challenging entity in interventional cardiology. CNs are characterized by irregular, protruding calcium masses that frequently lack a protective fibrous cap and are often located in areas of high mechanical stress, predisposing them to thrombosis and ACS, particularly ST-elevation myocardial infarction (STEMI) [32]. Some studies performed during classical autopsy findings suggest eruptive calcified nodules (Figure 5) with a concomitant thrombosis being the culprit of common cases of sudden cardiac arrest (SCA) and even death [33]. The use of advanced imaging techniques to assess calcium modification intravascularly during percutaneous coronary intervention (PCI) can also limit spatial resolution, depending on the specific imaging modality employed.

Intravascular ultrasound (IVUS) can provide information related to the arc, length, and superficial/deep texture of calcified plaques, but is restricted to the higher capabilities and superiority of optical coherence tomography (OCT), which can provide more than 10 times the spatial resolution of calcium deposits in the coronary walls when compared to the former imaging modality [33]. Both OCT and IVUS have demonstrated that CNs frequently present as eccentric, protruding calcific deposits disrupting luminal geometry. This morphology is associated with difficult wire passage, balloon slippage, and high rates of incomplete stent expansion, all of which translate into increased procedural complexity and suboptimal clinical outcomes [34,35].

Recent data further underscore the clinical significance of CNs as a driver of adverse outcomes and a marker of complex PCI. In a contemporary cohort of patients undergoing PCI for angiography and intravascularly identified CNs, we found that these lesions were frequently associated with high-risk clinical profiles—advanced age, diabetes mellitus, and chronic kidney disease—and demonstrated increased procedural challenges requiring advanced calcium modification techniques [36,37]. Notably, while traditional balloon-based or rotational strategies often yielded suboptimal lesion preparation, newer technologies have shown promise in improving procedural success and long-term outcomes in this difficult lesion subset. These findings highlight CNs as a critical focus in modern interventional cardiology, emphasizing the need for tailored strategies and device selection to optimize results.

The three subtypes of coronary artery calcified plaques that are the culprits of ACS include superficial calcific sheets, eruptive calcified nodules, and calcified protrusions, with superficial calcific sheets being the most common type, followed by eruptive nodules and lastly, calcified protrusions [38]. Based on angiographic findings post-percutaneous coronary intervention (PCI), it was discovered that eruptive calcified nodules were commonly located in the right coronary arteries (RCAs). In contrast, superficial calcific sheets were more common in the left anterior descending coronary arteries (LADs) [38]. A study conducted at Harbin Medical Center in China found that superficial calcific sheets were the sole culprits, leading to ACS in 69.4% of the total 258 calcified plaques analyzed through optical coherence tomography imagery, with 21.7% being eruptive calcified nodules and 8.9% being calcified protrusions [39].

Coronary calcium nodules (CNs) are often associated with diabetes mellitus and chronic kidney disease [32]. CN can be divided into two groups: eccentric calcium nodules (E-CNs) and non-eccentric calcium nodules (NE-CNs), with an E-CN being defined as a protruding calcified mass with fragments that can become disruptive to the fibrous cap [32]. Eccentric calcified plaques, characterized by asymmetric calcium deposition within the coronary artery wall, pose significant challenges during PCI due to their natural morphology and physical characteristics. Lastly, certain calcium plaques tend to grow longitudinally, either in a unidirectional or a bidirectional plane along the vascular lumen. The morphology of the growth appears to be horizontal rather than vertical and runs parallel to the vessel. This structural morphology will lead to a uniform decrease in lumen diameter rather than a stenotic lesion at one single point, and thus, the calcified plaque will grow outward.

An E-CN exhibits an irregular distribution, leading to areas of varying mechanical resistance, which complicates procedures such as balloon angioplasty and stent deployment [34]. Procedural challenges include resistance to balloon angioplasty; the rigidity of calcified segments can also impede balloon expansion, resulting in incomplete lesion preparation [34]. Stent deployment issues may arise from asymmetric calcification, which can result in the stent failing to fully adhere to the vessel wall, thereby increasing the risk of stent thrombosis and restenosis [34]. The presence of eccentric calcified plaques is associated with higher incidences of vessel dissection and perforation during PCI [35]. Aggressive post-dilation of stents to overcome the initially mispositioned struts can lead to vessel perforation, which can be avoided by utilizing a prolonged balloon inflation technique to combat and prevent vessel perforation [35].

## 5. Risk Factors and Prevalence

The landmark Multi-Ethnic Study of Atherosclerosis (MESA), which was initiated in 2000 as an interdisciplinary analysis study between research titans within the National Heart, Lung, and Blood Institute (NHLBI), created the framework to understand the prevalence, progression, determinants, and prognostic significance of subclinical cardiovascular disease in a sex-balanced, multiethnic, and community-dwelling U.S. cohort [40]. The birth of the Framingham Heart Study in Massachusetts in the late 1940s became a prime example of efforts to identify primary traditional atherosclerotic cardiovascular disease (ASCVD) risk factors, such as high cholesterol, high blood pressure, smoking, and diabetes, as predictors of morbidity and mortality from cardiovascular disease.

Age is another predictor of cardiovascular disease, as asymptomatic patients from four major ethnically rich groups (White, Black, Hispanic, and Chinese) across six US cities underwent serial CT scans to determine calcium scoring every 2 years, with detection seen at 12% amongst those aged > 80 and only 5% amongst those aged < 50 [41]. Males were also seen as having a more frequent coronary calcification burden when compared to females, as it is likely that hormonal protection against atherosclerosis exists through the physiological mechanisms of estrogen [41]. The findings of the CRIC study, which examined 1100 patients aged between 21 and 74 years with mild-to-moderate chronic kidney disease (CKD) and without known coronary artery disease (CAD), who underwent CT coronary artery calcium scoring, resulted in 60% having calcified CAD, strengthening the relationship between complications related to CKD and CAD [42].

Researchers from Japan investigated the prevalence of coronary artery calcification amongst their population when compared to Western individuals. They discovered that the Japanese lifestyle could contribute to cardiovascular disease, mainly when analyzed through coronary artery calcium scores using CT imaging. The study took 201 patients aged > 20 through a series questionnaire asking them about their lifestyle, medical history, and other factors, with the results showing that individuals who smoked, had sedentary work, and short sleep times had a higher odds ratios than those that consumed Japanese-style breakfast (e.g., boiled rice, miso soup, and grilled fish), who had a lower odds ratio association [43].

## 6. Role of Imaging

The usage and value of intracoronary imaging modalities in guiding revascularization during PCI procedures, where calcium modification creates a significant challenge, are notable. Extensive coronary artery calcification lesions can negatively impact PCI outcomes and are associated with a higher incidence of “stent under-expansion,” where the stent fails to expand adequately because of lumen sclerosis caused by large calcium plaques.

Without the utilization of advanced intraoperative imaging studies during balloon dilation in PCI procedures, the risk of high-pressure dilation between the calcium deposition and the soft-tissue matrix within the coronary vasculature can lead to disruption, result in dissection, and even compromise the structural integrity of the device during the process of stent deployment [44].

A study involving more than 19,000 patients who received PCI and had prior moderate-to-severe calcified coronary artery stenosis before the intervention still had a higher rate of major adverse events, which included an increased rate of cardiac death and stent thrombosis, when compared to the group of patients who had noncalcified lesions and underwent PCI [45].

**Figure 6:**
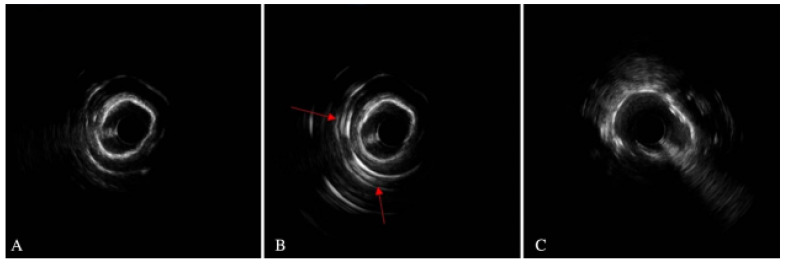
IVUS-guided rotational atherectomy (RA). (**A**) Pre-PCI imaging illustrating heavy calcified plaque with a 360-degree calcium arc. (**B**) After RA utilizing a 1.5 mm bur, with arrows showing reflection posterior to the calcification. (**C**) After PCI stent implantation with high-pressure post-dilatation in the setting of IVUS, showing persistent stent under-expansion. IVUS = intravascular ultrasound; RA = rotational atherectomy; PCI = percutaneous coronary intervention. Source: Teng, W.; Li, Q.; Ma, Y.; Cao, C.; Liu, J.; Zhao, H.; Lu, M.; Hou, C.; Wang, W. Comparison of optical coherence tomography-guided and intravascular ultrasound-guided rotational atherectomy for calcified coronary lesions. *BMC Cardiovasc. Disord.* **2021**, *21*, 290.

**Figure 7:**
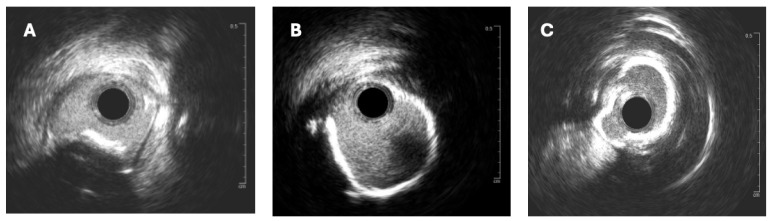
IVUS imagery: (**A**) Calcified nodule (7 o’clock); case involving IVL RCA. (**B**) 360-degree proximal superficial calcium plaque. (**C**) Case of patient with LCx modification who had history of ESRD; post-IVL with multiple sites of calcium fracture (7 o’clock and 11 o’clock, superficial and deep calcium plaques). RCA = right coronary artery; LCx = left circumflex coronary artery; ESRD = end-stage renal disease. Source: KaChon Lei, M.D.

**Figure 8:**
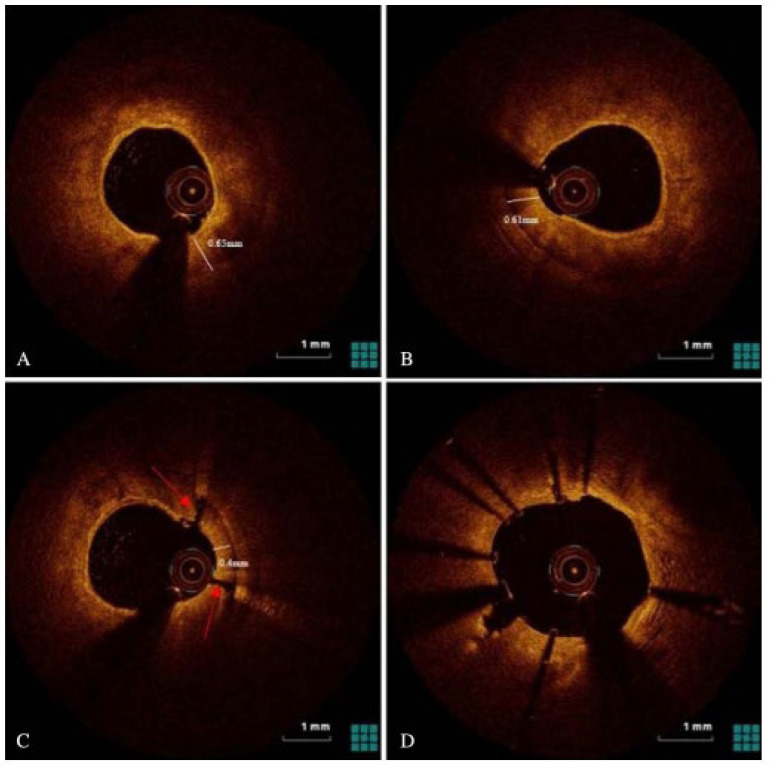
Imagery of optical coherence tomography (OCT)-guided rotational atherectomy (RA). (**A**) Phase of pre-PCI OCT images depicting heavy plaque calcification with a 360-degree calcium arc and minimal calcium thickness of 0.65 mm. (**B**) Post-RA step utilizing a 1.5 mm burr with calcium thickness measuring 0.61 mm without any obvious crack formation in plaque structure. (**C**) Upsizing the burr tip to 1.75 mm, calcium thickness became 0.40 mm with apparent cracks forming (red arrows) in the calcium plaque. (**D**) After stent implantation with post-dilatation, utilizing OCT imagery detailing successful stent expansion. Source: Teng, W.; Li, Q.; Ma, Y.; Cao, C.; Liu, J.; Zhao, H.; Lu, M.; Hou, C.; Wang, W. Comparison of optical coherence tomography-guided and intravascular ultrasound-guided rotational atherectomy for calcified coronary lesions. *BMC Cardiovasc. Disord.* **2021**, *21*, 290.

**Figure 9:**
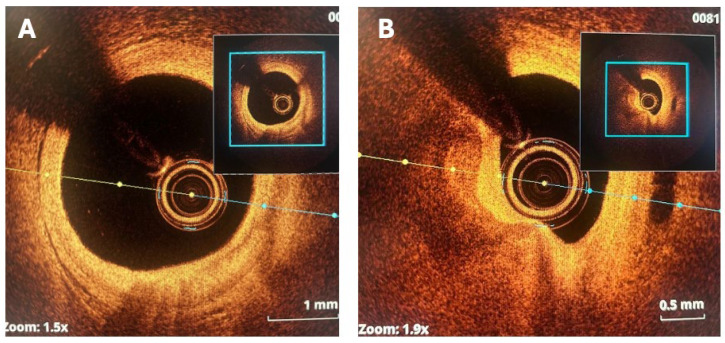
OCT imagery. (**A**) Superficial and deep calcium plaques at the 4 and 6 o’clock positions. (**B**) Obstructive disease with non-eruptive calcium nodule and deep calcium plaque on OCT between the 6 and 11 o’clock positions. Source: KaChon Lei, M.D.

The effective execution and operation of intracoronary imaging modalities during these procedures improve both procedural and long-term clinical outcomes, particularly in intravascular ultrasound (IVUS) (Figure 6 and Figure 7) and optical coherence tomography (OCT) (Figure 8 and Figure 9), where coronary lesion morphology and mapping facilitate better PCI procedural planning [46]. Both IVUS and OCT can detect, localize, and quantify coronary calcification. Notably, OCT can visualize the calcified plaque without producing visual imaging artifacts, thereby minimizing observational scatter, and can evaluate calcium thickness more accurately than IVUS.

A 3D calcium evaluation displaying a color-coded visual depiction of calcified plaques utilizing coronary CT imagery is a remarkable feat that analyzes the calcium plaque’s location relative to the circumference of the coronary vessels while computing the location of the vessel wall in relation to the superior (epicardial) or inferior (myocardial) borders [44]. The 3D calcium evaluation through CT imagery is a tool that gauges the coronary tree’s curvature, tortuosity, and radius while assisting in predicting the path of the guide wire to prevent or minimize contact with calcium plaque deposits within the vessel.

### 6.1. Coronary CT Angiography

**Figure 10:**
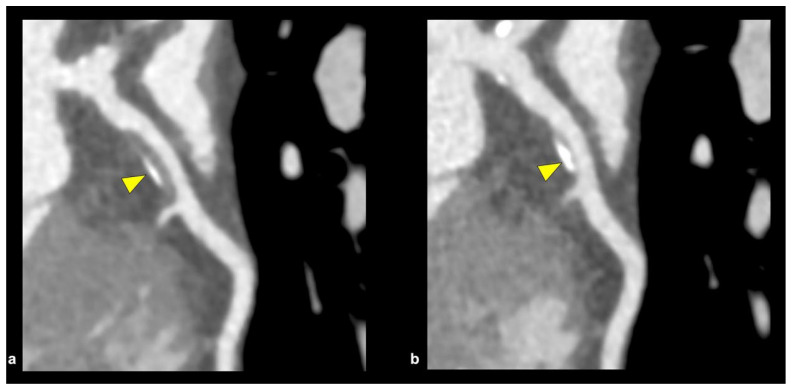
Morphological physical change in coronary artery plaque seen on CCTA, showing a partially calcified plaque in the proximal LAD artery. (**a**) The arrowhead depicts plaque size prior to the initiation of optimal medical therapy. (**b**) The arrowhead depicts coronary plaque has downsized and the calcified portion has increased after 3 years on optimal medical therapy. CCTA = coronary computed tomography angiography; LAD = left anterior descending. Source: Yoshida, K.; Tanabe, Y.; Hosokawa, T.; Morikawa, T.; Fukuyama, N.; Kobayashi, Y.; Kouchi, T.; Kawaguchi, N.; Matsuda, M.; Kido, T.; et al. Coronary computed tomography angiography for clinical practice. *Jpn. J. Radiol.* **2024**, *42*, 555–580.

Coronary CT angiography (CCTA) (Figure 10) is a non-invasive tool that provides high sensitivity and high spatial resolution. It quantifies the coronary artery calcium score (CACS) for the long-term assessment, risk stratification, and treatment of high-risk plaque features, which include positive remodeling, low attenuation, the “napkin-ring sign,” and spotty calcification [47,48]. The main culprit and high-risk lesion in ACS patients are the presence of the “spotty pattern” of calcium deposit plaques (focal calcification < 3 mm in diameter) when compared to the non-culprit calcium lesion (contiguous calcification > 3 mm in diameter), but discrepancies and inconsistencies occur when the highly dense calcium plaques (>1 K Hounsfield units on CCTA) are included [48].

The Incident Coronary Syndromes Identified by Computed Tomography (ICONIC) study elaborated that highly dense calcium plaques analyzed by CCTA were associated with stable disease and lowered the risk of ACS based on per-patient and per-lesion calculations, which were implemented and supported in previous studies, such as the PARADIGM study that suggested statin therapy was associated with increased calcification burden but reduced necrotic core volume and thereby lowering the overall risk of ACS [49,50]. The ICONIC study is in conflict with newer studies that have suggested that a CAC > 1 K has a higher all-cause and cardiovascular disease (CVD) mortality. This suggests that highly dense plaques, with the understanding of the CAC score (combination of CAC density and CAC volume), allude to the fact that isolated higher CAC density may be a marker of stable plaque, but higher CAC volume signifies more plaque burden and higher CVD risk [51].

To reduce partial volume and beam-hardening artifacts, high-spatial-resolution CCTA scanners have been developed with higher in-plane spatial resolution (0.2–0.23 mm) compared to conventional 64-section multidetector CT, which typically has a standard definition of 0.5–0.75 mm [48]. High-spatial-resolution CCTA, when compared with standard-resolution CCTA, improves the assessment, accuracy, and overall evaluation of calcified lesions, as well as regions of atheroma and positive arterial remodeling, which can lead to calcified plaques provoking ACS [52].

CCTA images can be utilized to assess and calculate intravascular calcium burden and to measure the risk of future heart disease. The Agatston Calcium Score Interpretation, sometimes referred to as the coronary artery calcium (CAC) score, has garnered great interest from clinicians in the detection and management of CAD [53]. Interpretations of coronary artery calcium (CAC) score results are based on the Agatston method and are categorized on values of 0: no evidence of CAD, 1–100: mild evidence of CAD, 101–400: moderate evidence of CAD with increased risk of future coronary events, and lastly, >400, equating to severe evidence of CAD, which places individuals at high risk of myocardial infarction due to increased calcium plaque burden (Table 1) [54]. In certain studies, there is a more specific delineation between minimal and mild cases of CAD based on CAC scores, such that 1–10 constitutes a minimal disease state versus 11–100 signifying mild disease.

Complex calcified plaques place a huge burden on the coronary arteries in such a way that partial or even complete chronic total occlusions (CTOs) of the native vessel can occur, thereby greatly reducing the overall diameter of the vessel to far below the normal hemodynamic flow it needs. “Full-moon” coronary calcification occludes the entire coronary vessel and is detected through CCTA imagery [55]. This plays an important role in detecting vascular architecture and the procedural planning needed for calcium plaque debulking. The “full-moon” calcification that was detected by CCTA was studied in two European centers, and after analysis of 1324 CTO-PCIs was performed, it was found that CTOs in association with “full-moon” calcific lesions were more commonly seen in proximal locations in coronary vessels, in addition to a high dominance in the right coronary artery [55]. CCTA has played a crucial role in being able to examine, assess, and procedurally plan complex calcium plaque, like CTO with “Full moon” morphology.

### 6.2. Positron Emission Tomography

**Figure 11:**
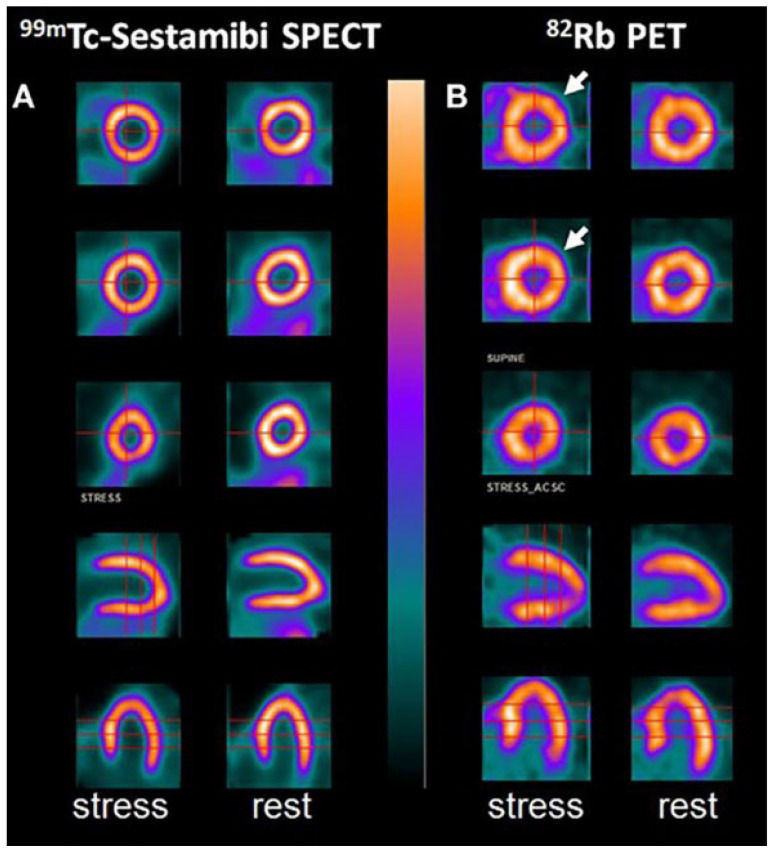
(**A**) ^99m^Tc-Sestamibi SPECT images in both stress and rest state. (**B**) ^82^Rb PET images in both stress and rest state. ^82^Rb-PET images depict ischemia involving the circumflex artery (arrow), shown after dipyridamole administration. ^99m^Tc = radioactive isotope technetium-99 m; SPECT = single-photon emission computed tomography; ^82^Rb = radioactive isotope rubidium-82; PET = positron emission tomography. Source: Chatal, J.F., Rouzet, F.; Haddad, F.; Bourdeau, C.; Mathieu, C.; Le Guludec, D. Story of rubidium-82 and advantages for myocardial perfusion PET imaging. *Front. Med.* **2015**, *2*, 65.

The high spatial resolution and accuracy that imaging studies such as positron emission tomography (PET) (Figure 11) have, while utilizing fluorine isotopes to identify high-risk calcium plaques earlier in their progression, is better than CCTA because of the lower thresholds (200–500 µm) that the PET scan can operate on [48]. There is a relationship between ^18^F-NaF uptake and coronary calcification progression in stable CAD, as this molecular isotope represents a strong predictive marker for coronary artery calcification [56].

In addition to coronary artery disease, PET imaging utilizing ^18^F-NaF uptake has also been introduced in other vascular studies to examine other physical morphologies more effectively, including carotid atherosclerosis, abdominal aortic aneurysm (AAA) disease, aortic stenosis, bioprosthetic valve degeneration, and erectile dysfunction [57]. Lastly, the prognostic value of ^18^F-NaF uptake using PET-CT to assess coronary artery calcified plaques is currently being investigated through the ongoing prospective study, pioneered by the Prediction of Recurrent Events With ^18^F-Fluoride (PREFFIR) study [48].

### 6.3. Photon-Counting CT and Dual-Energy CT

**Figure 12:**
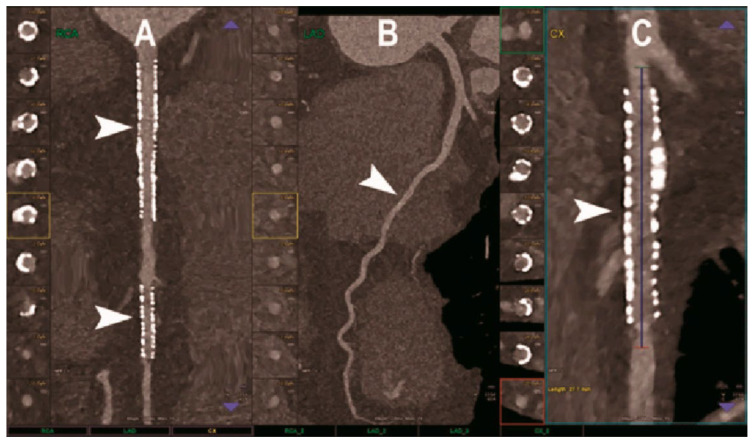
PCCT depicting multiplanar reconstruction of a coronary artery tree. (**A**) Image illustrates the RCA with 2 stents (arrows), one being proximal (larger) and one distal (smaller). (**B**) The LAD coronary artery elongated across the LV apex, coursing adjacent to the intramyocardial zone in the middle segment of the vessel (arrow). (**C**) The left circumflex coronary artery depicting a stent with adequate intrastent visualization and patency (arrow). PCCT = photon-counting computer tomography; RCA = right coronary artery; LAD = left anterior descending; LV = left ventricle. Source: Meloni, A.; Frijia, F.; Panetta, D.; Degiorgi, G.; De Gori, C.; Maffei, E.; Clemente, A.; Positano, V.; Cademartiri F. Photon-counting computed tomography (PCCT): technical background and cardio-vascular applications. *Diagnostics* **2023**, *13*, 645.

Photon-counting CT (PCCT) (Figure 12) provides significantly superior imaging and diagnostic confidence in addition to the high quality of spatial resolution, soft-tissue contrast, and radiation dose efficiency while offering reduced partial volume averaging, better morphological depiction of CAC, and lower imaging noise [48,58]. Recent research has also shown that PCCT images have fewer blooming artifacts, less volume overestimation compared to micro-CT studies, and greater volume quantification accuracy with improved diagnostic quality of coronary calcifications [59,60].

**Figure 13:**
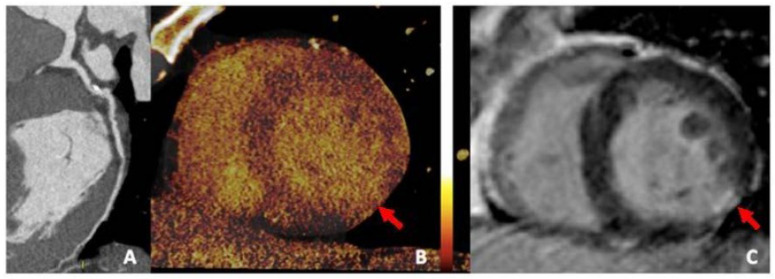
DECT of the left circumflex coronary artery. (**A**) Image depicting severe proximal stenosis due to the presence of calcified plaque inside the left circumflex coronary artery. (**B**) Iodine perfusion mapping demonstrating a defect in the inferolateral wall (arrow) corresponding to a prior myocardial scar (arrow). (**C**) Necrosis (arrow) caused by persistent myocardial ischemia of the inferolateral wall. DECT = dual-energy CT. Source: Dell’Aversana, S.; Ascione, R.; De Giorgi, M.; De Lucia, D. R.; Cuocolo, R.; Boccalatte, M.; Sibilio, G.; Napolitano, G.; Muscogiuri, G.; Sironi, S.; et al. Dual-energy CT of the heart: a review. *J. Imaging* **2022**, *8*, 236.

Dual-energy CT (DECT) (Figure 13), also known as spectral CT, is another emerging imaging technique that provides high-quality anatomic information on CAC while enhancing plaque visualization and facilitating the accurate assessment of high-risk calcified plaques by combining information from both CT and the effective atomic number [61]. DECT enables the use of multiple virtual monoenergetic images to reduce blooming artifacts caused by highly dense calcifications, and it facilitates intracavity visualization by decreasing background noise, which is a limitation of CCTA [48,62].

PCCT possesses the intrinsic value and capability of computing and classifying photons, enabling spectral imaging to achieve higher spatial resolution while incorporating its advanced devices into cardiac-designed CT scanners [48]. This will lead to augmentation of a dual-source CT scanner with superior temporal resolution of approximately 66 milliseconds compared to an inferior single-source CT scanner with a temporal resolution of 120–125 milliseconds, which, when compared, will drastically reduce residual motion artifacts, leading to a more crisp, accurate, and precise physical morphology assessment of the coronary artery calcified plaques within the intraluminal vasculature [48].

### 6.4. Balloon Escalation Technique

**Figure 14:**
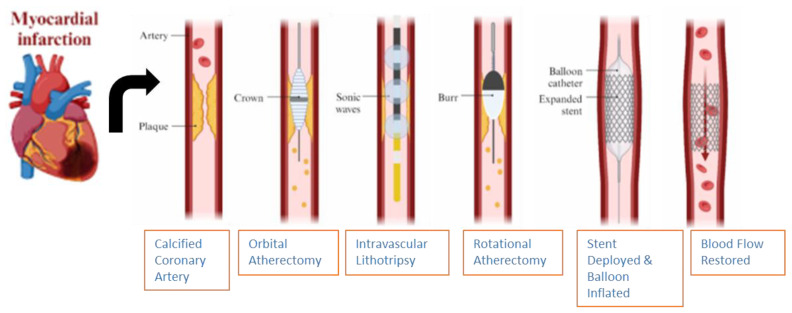
Mechanisms of action for various tools utilized in calcium modification techniques in PCI treatment of coronary artery calcified plaques. PCI = percutaneous coronary intervention. Adapted: Florek, K.; Bartoszewska, E.; Biegała, S.; Klimek, O.; Malcharczyk, B.; Kübler, P. Rotational atherectomy, orbital atherectomy, and intravascular lithotripsy comparison for calcified coronary lesions. *J. Clin. Med.* **2023**, *12*, 7246.

The various options provided by intravascular imaging techniques facilitated by mechanical modifications of calcified plaque deposits in the coronary vessels are vast and abundant (Figure 14). Conventional balloon angioplasty (BA), using either semi-compliant or non-compliant balloons (NCBs), is effective in modifying coronary lesions before stent implantation, thereby achieving the goal of minimal stent-area management [46]. Concentrically calcified lesions within the coronary arteries can cause non-uniform balloon expansion, even in the setting of utilizing NCBs in high-pressure modes, leading to the visual phenomenon termed “dog-bone deformity” [46]. This visual appearance is caused when relative under-expansion occurs within the center calcified segment compared to the outside edges of the calcified segment.

**Figure 15:**
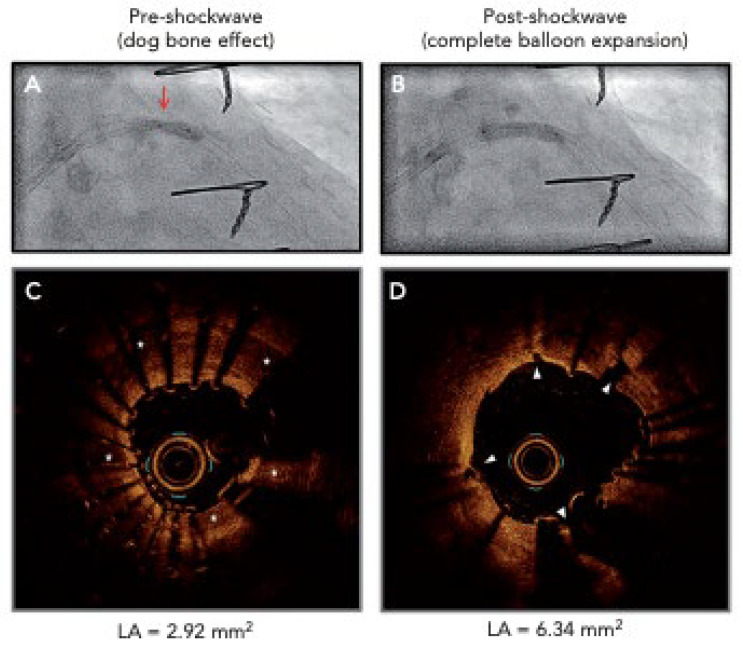
(**A**) Before IVL, with red arrow showing “dog bone” deformity; (**B**) after IVL, showing an increasing size of the coronary vessel attributed to mechanical calcium modification; (**C**) OCT before IVL, with asterisks showing calcium deposits in the coronary vessel walls; (**D**) after IVL device application, with arrows showing calcium fracture. IVL = intravascular lithotripsy; OCT = optical coherence tomography; LA = vascular lumen area. Red arrows, asterisks, & white arrows have been described under the figure description. Source: Forero, M.N.T.; Daemen, J. The coronary intravascular lithotripsy system. *Interv. Cardiol. Rev.* **2019**, *14*, 174.

The “dog-bone deformity” (Figure 15) leads to inadequacies of lesion preparation, which can ultimately lead to balloon rupture, vessel dissection, and, in severe cases, coronary artery perforation. Despite NCB’s characteristics of enduring high pressures of approximately 20–24 atm, the single-layer structures of the balloon make them susceptible to damage from calcium plaques, especially if the NCBs are single-layered and spiculated [46].

Scoring and cutting balloon devices with a semi-compliant or non-compliant variation type surrounded by an external helical scoring edge can be indicated in proximal and distal lesions, aorto-ostial lesions, tortuous coronary segments, and after rotational atherectomy (RA), orbital atherectomy (OA), or intravascular lithotripsy (IVL) [44]. The device itself creates three to four endovascular radial incisions through the fibrocalcific tissue segment using a scoring/cutting mechanism, which further allows for expansion and dilation compared to traditional balloons [44].

### 6.5. Intravascular Lithotripsy

Intravascular lithotripsy (IVL) (Figure 15) is a minimally invasive procedure that utilizes acoustic shockwaves by generating high-amplitude ultrasonic pressure waves that vaporize the saline/contrast mixture within the balloon, causing it to induce circumferential calcium micro-fractures and fissures present within both the intimal and medial layers of the lumen [46,63]. The IVL catheter consists of an approximately 0.014-inch guidewire that is compatible with a balloon angioplasty catheter with two spark gap-based lithotripsy emitters incorporated into the shaft of the 12 mm-long balloon, and its indications to assess deep calcification and calcified nodules, large vessels, stent under-expansion (off-label), bifurcation lesions, and calcified aorto-ostial lesions are utilized [44]. 

The limitations of IVL imaging include its high cost, which restricts its use in various countries, particularly those in the developing world. According to the Centers for Medicare & Medicaid Services (CMS), the national average cost of inpatient hospital payment for IVL in 2023 was USD 20,785 for coronary IVL and USD 12,450 for peripheral IVL. Cross-over ability and utilization of the probes between intravascular ultrasounds (IVUS) and optical coherence tomography (OCT) through the complex geographical mapping of intraluminal calcified plaques can also be challenging [64]. Due to their larger size profile, IVL balloons may be unsuccessful or have difficulty navigating through tight coronary lesions, potentially leading to obstructions that can be resolved by utilizing the rotational atherectomy technique before engaging IVL [65].

The clinical outcomes of IVL when compared to RA are superior in the aspect that IVL does not increase the risk of coronary perforation and slow flow or abrupt vessel closure [66]. IVL is one of the most premier devices due to its smaller size profile, the simpler design, and ergonomic comfortability [66]. IVL is mostly utilized on circumferential calcium plaques, but recent exploration for the use of calcified nodules (CNs) is currently being investigated [66]. The Disrupt CAD I, II, III, and IV study trials have confirmed the safety and effectiveness of IVL for the treatment of severely calcified lesions while exhibiting high procedural success with minimal complications over general atherectomy devices [66]. A comprehensive analysis of all four Disrupt CAD trials showed successful stent implantation with a high procedural success rate of 92.4% and freedom from major adverse cardiovascular events (MACEs) at 30 days at a rate of 92.7% [66].

The device challenge of IVL during peri-procedural PCI is the slight tendency to cause “shockotopics,” a term for transient IVL-induced capture leading to ventricular ectopic activity [66]. This occurs when the acoustic pressure waves depolarize the cardiac tissue via the stretch-activated response, which is often generated by IVL, and the heart rate is lower than 60 beats/min [66]. This interesting phenomenon was seen in 41.1% of cases in the Disrupt CAD III cohort, but overall, it did not lead to a sustained ventricular arrhythmia of any clinical significance [66]. Further investigations concluded that the 8 μJ of mechanical energy applied as a result of the IVL balloon was several magnitudes lower than the electrical energy needed to induce ventricular tachycardia or ventricular fibrillation, which is often in the range of 0.6–2.0 J [66].

### 6.6. Rotational Atherectomy

**Figure 16:**
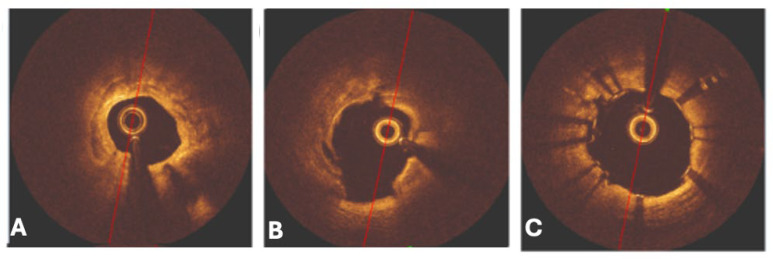
OCT imaging scans representing the RA calcium modification technique. (**A**) OCT imagery showing a cross-sectional plane of severely calcified coronary artery stenosis. (**B**) After RA technique application, showing intima dissection at 8–9 o’clock and a calcified plaque fracture at the 12 o’clock position. (**C**) Final result post-RA and stent implantation of the same coronary artery in prior images, noting the increase in luminal size when compared to pre-RA intervention. Red line = represents clock face dial with top of the line representing 12 o’clock and the bottom line representing the 6 o’clock position; OCT = optical coherence tomography; RA = rotational atherectomy. Adapted: Blachutzik, F.; Meier, S.; Weissner, M.; Schlattner, S.; Gori, T.; Ullrich, H.; Gaede, L.; Achenbach, S.; Möllmann, H.; Chitic, B.; et al. Coronary intravascular lithotripsy and rotational atherectomy for severely calcified stenosis: Results from the ROTA. shock trial. *Catheter. Cardiovasc. Interv.* **2023**, *102*, 823–833.

Both atherectomy techniques use either a rotational atherectomy (RA) (Figure 16) or orbital atherectomy (OA) to rapidly induce the breakdown of calcified plaques inside the coronary arteries, utilizing a crown or burr. The RA device, which was first introduced over 30 years ago, contains a diamond-encrusted elliptical burr called a rotablator that moves at high speeds ranging from 140,000 to 160,000 rpm and can even rise as high as 190,000 rpm, which then pulverizes the calcified plaques in a forward direction [67,68].

The limitations and risks of RA include prolonged usage, which can lead to higher temperatures and increased heat generation, potentially causing artery perforation, microembolization, or vessel dissection, in addition to avoiding decelerations above 5000 rpm [68]. The use of heparin, Rota Glide™ lubricant, and vasodilators is recommended to help prevent possible vasospasms, lower generated heat, and no-/slow-flow complications, which can be managed more effectively by utilizing smaller burrs at lower speeds, thereby reducing the risk of such complications [68]. Other limiting factors that contribute to the discouraged utilization of this device include the lack of standardized protocols, insufficient structured training, and concerns about procedural complexity and potential complications [67].

Comparisons of safety profiles between IVL and RA include differences between complication rates for the treatment of severe coronary artery calcification. A cross-sectional observation trial conducted in a single center included 152 and 238 patients who underwent IVL and RA, respectively, between January 2023 and November 2023. The results of this study concluded that procedural complications and major adverse cardiac events were more pronounced in cases involving RA [69]. These procedural complications included perforation, cardiac tamponade, flow-limiting dissection, major adverse cardiovascular events (MACEs), non-fatal MI, target vessel revascularization, and all-cause mortality.

Studies have shown that RA can be safely utilized to treat under-dilated and under-expanded stents [70]. When compared to IVL, RA needs to utilize an over-the-wire system that employs a burr connected to a drive shaft, flush solution, and rotablation console, making the procedure very technically challenging [70]. The IVL setup consumes fewer resources, such as cath-lab time and materials, and reduces overall procedural complexity [70]. Outcomes between RA and IVL are nearly the same at the hands of a proficient operator, and no difference in all-cause death exists [71]. When measuring for perioperative complication endpoints, both RA and IVL have similar safety profiles, including low risk of cardiac death, procedurally related MI, CVA, coronary perforations, severe coronary dissection, no reflow, vascular access complications, and emergent coronary artery bypass grafts (CABGs) [70]. 

In a study conducted in two European medical centers, researchers compared in-stent pressure gradients assessed by vessel fractional flow reserve (vFFR) in coronary artery calcified plaque lesions treated with either RA or IVL. From a cohort of 210 patients with 105 matched individuals, amongst 70 patients in the RA group and 35 in the IVL group, post-analysis after a PCI procedure showed that in-stent pressure gradients were significantly lower in the IVL group [72]. Researchers discovered that treatment with IVL for calcium modification during PCI provided the foundation to have better stent expansion compared to RA, with higher FFR values associated with better prognosis than low FFR values post-PCI, suggesting plaque modification was more effective with IVL than with RA [72].

### 6.7. Orbital Atherectomy

**Figure 17:**
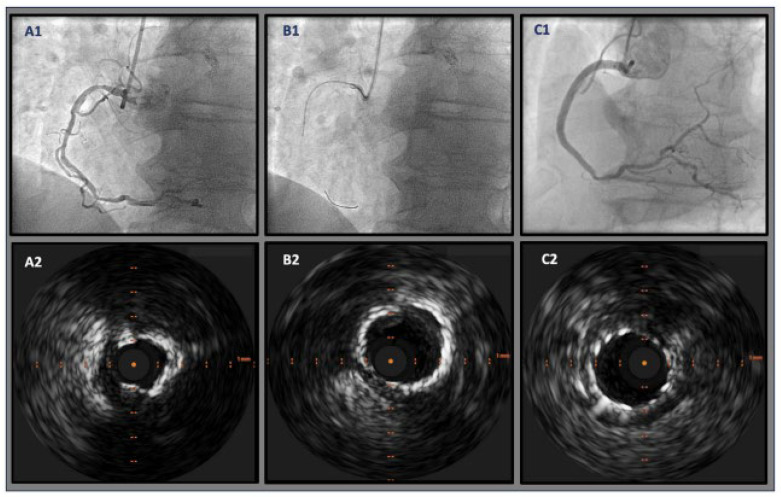
IVUS-guided illustration. OA-facilitated PCI with calcium modification technique of the RCA, supported by angiography imagery. (**A1**) Severe diffuse calcified lesion on the proximal and mid-segments of the RCA; (**A2**) severe 360-degree calcified lesions of the RCA; (**B1**) OA placed inside the mid segment of the RCA, with subsequent advancements and retreatments of the mechanical device having been made; (**B2**) reassessment post-OA showing beneficial sanding through echo reverberations and luminal gain; (**C1**) final angiographic assessment post-PCI stenting of the RCA from the mid-distal segment through to the ostium; (**C2**) final IVUS-guided imagery showing post-PCI stenting with adequate apposition and expansion of the stent in the lumen of the RCA. IVUS = intravascular ultrasound; OA = orbital atherectomy; PCI = primary coronary intervention; RCA = right coronary artery. Source: Faria, D.; Vinhas, H.; Bispo, J.; Guedes, J.; Marto, S.; Palmeiro, H.; Franco, P.; Mimoso, J. Initial experience with orbital atherectomy in a non-surgical center in Portugal. *Rev. Port. De Cardiol.* **2024**, *43*, 659–665.

Orbital atherectomy (OA) (Figure 17) features a small eccentrically mounted diamond-coated crown that electrically operates a drive shaft, spinning the crown up to 80,000–120,000 rpm through centrifugal forces, which are exerted inside the lumen of the vessel to smoothen the thin layer of calcified plaque by ablating inelastic calcified plaque while preserving compliant tissue and deflecting away softer tissues [67]. Unlike rotational atherectomy (RA), OA’s advantage includes the athero-ablation function, which is bidirectional, thereby reducing the risk of device entrapment and providing a circumferential plaque modification, which is particularly advantageous in lesions with eccentric or protruding calcium.

A commonly seen complication with rotational atherectomy (RA) is the creation of a large debris burden after plaque destruction and remodeling. By generating micro-sized particulate debris with a smaller footprint it possesses from the calcium plaques, it sands down, and OA is advantageous over RA because the calcium pieces that are removed from this mechanical technique are approximately 2 µm (smaller than an RBC), resulting in lower rates of heart block and reducing the risk of distal embolization, an important consideration in complex lesions [67]. OA disadvantages include the use of a single 1.25 mm crown size, which limits its usage but can be optimized and overcome at higher rpm settings [67]. 

These characteristics make OA especially suited for calcified nodules (CNs), one of the most technically demanding subsets of calcified coronary disease. CNs’ irregular protruding morphology frequently results in inadequate lesion preparation and suboptimal stent expansions when using conventional strategies, contributing to restenosis and stent thrombosis. OA’s ability to create a more uniform luminal architecture, minimize wire bias, and avoid device entrapment has positioned it as a valuable technique for these lesions. Recent studies have demonstrated high procedural success, low peri-procedural complication rates, and favorable mid-term outcomes with OA in the treatment of CNs [36,37]. These findings support OA as an effective and reproducible strategy for optimizing lesion preparation in complex calcified lesions, particularly those with nodular morphology, and highlight its expanding role in contemporary PCI.

Studies have shown that PCI of calcified plaques in coronary arteries is associated with poor outcomes due to a lack of adequate stent delivery, disruptions in drug-carrying polymers, and impairments in physical kinetics, which lead to an under-expanded stent (UES) [66]. The most common complication post-PCI, because of acute stent thrombosis and in-stent restenosis, is caused by the initial UES step. Recent developments have discovered that both RA and OA have improved clinical outcomes in the setting of PCI with concomitant severely calcified lesions being present, but this positive outcome is dependent on operator mastery [66]. This presents a significant challenge, which causes a steep learning curve in accordance with peri-procedural complications should the operator lack adequate experience and be under-proficient in utilizing RA and OA devices during PCI [66].

### 6.8. Laser Atherectomy

**Figure 18:**
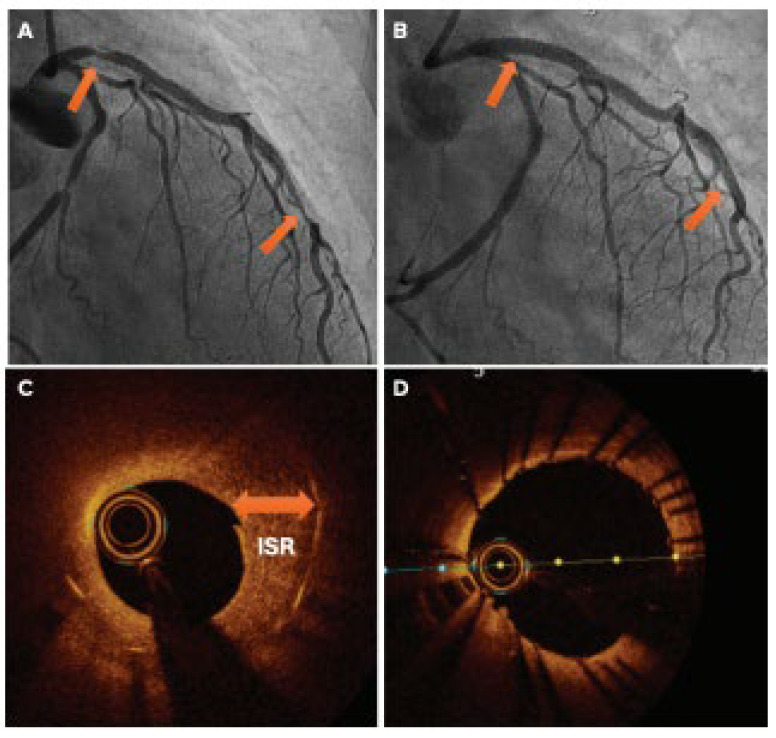
(**A**) Angiography imagery showing a severe calcified lesion in the proximal and distal LAD coronary artery (arrows). (**B**) Angiography illustration of post-LA utilization supported by (**C**) OCT imagery of the same segment pre-treatment with LA and (**D**) post-treatment with LA. LAD = left anterior descending; LA = laser atherectomy; OCT = optical coherence tomography; ISR = in-stent restenosis. Source: Khattak, S.; Sharma, H.; Khan, S.Q. Atherectomy techniques: rotablation, orbital and laser. *Interv. Cardiol. Rev. Res. Resour.* **2024**, *19*, e21 [67].

Laser atherectomy angioplasty, also known as excimer laser coronary angioplasty (ELCA) (Figure 18), gained popularity shortly after its development in the late 1980s, utilizing pulsatile laser energy and short wavelengths for the precise ablation of calcified plaques without the risk of thermal injury and burns to surrounding vessels [67]. Light amplification by stimulated emission of radiation (LASER) utilizes a high-energy single-wavelength light beam from a gas mixture. It is the foundation of how ELCA functions, by mixing xenon gas and diluted hydrogen chloride in a solution to create a high-energy laser beam that can modify and break down the calcified plaques in coronary arteries [67].

Initially, ELCA showed higher complication rates, including vascular dissections related to significant ischemic changes and perforation. However, with improvements in technology, operator experience, patient selection, and modifications to device engineering and design, complication rates have significantly decreased over the last decade [67]. The LEONARDO study in 2015 and the subsequent ELCA UK registry study found no significant association between ELCA use and coronary perforations in a diverse patient cohort [73]. The ROLLER-COASTER trial was the first randomized comparison of RA, ELCA, and IVL to be utilized in moderate-to-severe calcified lesions and showed similar rates of success in mean stent area (MSA) across all three procedural devices [74].

In one retrospective study including 21,256 patients undergoing PCI over the span of 16 years (April 2005–May 2021), with 448 (2.1%) of those undergoing treatment with ELCA, showed that ELCA in PCI was associated with a higher frequency of any peri-procedural complications [75]. However, the study concluded that the usage of ELCA PCI was utilized as a last-ditch effort in higher risk populations with complex CAD and elevated calcium plaque burden, and thus, there was no direct and significant long-term increased mortality associated with use of the ELCA device [75].

Positive outcomes pertaining to ELCA PCI include the greater achievement of attaining stent expansion at higher rates and long-term durable mechanics if the risk of restenosis is, in part, due to stent under-expansion [76]. An advantage of ELCA over other atherectomy devices in situations where there is heavy calcification behind an already existing stent strut, includes the ability of ELCA to modify calcium architecture better than balloon angioplasty while under the guise of OCT imagery [76]. Thus, ELCA PCI does have some useful and achievable advantages and positive outcomes over other calcium-modifying devices, including IVL and atherectomy devices.

## 7. Conclusions

Calcium modification in percutaneous coronary intervention remains a vast and elaborate field that is continuing to engineer, develop, and refine technical practices and niche mechanics to remodel atherosclerotic calcified plaques that, if left untreated, can lead to devastating outcomes such as myocardial infarctions, deadly arrhythmias, sudden cardiac arrest, and even death. The benefits of cardiac imaging, including the utilization of IVUS, OCT, CCTA, PET, PCCT, and DECT, enable the operating cardiologist to better visualize coronary artery calcium plaque burden, which is much like being able to see the world in complete darkness. It enables the operator to facilitate further procedures and execute modern applications, such as balloon angioplasty, intravascular lithotripsy, rotational atherectomy, orbital atherectomy, laser atherectomy, and, ultimately, stent placement, to achieve their goal of decreasing plaque burden, leading to life-saving measures that reduce morbidity and mortality for patients. This comprehensive review represents a robust and detailed organizational framework that greatly contributes to the field of interventional cardiovascular medicine by providing the analysis and therapeutic options to manage complex coronary artery calcified plaques and positively impacts the ongoing literature and field of study.

## Figures and Tables

**Table 1:** The interpretation of the coronary artery calcium (CAC) score and CT findings.

Visual Score	Absolute CAC Score (Agatston Method)	Clinical Interpretation
None	0	Very low risk of future coronary events
Minimal	1–10	Low risk of future coronary events; low probability of MI
Mild	11–100	Medium risk of future coronary events
Moderate	101–400	Increased risk of future coronary events; consider reclassifying the individual as being at high risk
Severe	>400	Increased probability of MI

MI = myocardial ischemia.

## Data Availability

No new data were created or analyzed in this study.
